# Modern geographical reconnaissance of target populations in malaria elimination zones

**DOI:** 10.1186/1475-2875-9-289

**Published:** 2010-10-20

**Authors:** Gerard C Kelly, Jeffrey Hii, William Batarii, Wesley Donald, Erick Hale, Johnny Nausien, Scott Pontifex, Andrew Vallely, Marcel Tanner, Archie Clements

**Affiliations:** 1 Pacific Malaria Initiative Support Centre, Australian Centre for International and Tropical Health, School of Population Health, University of Queensland, Brisbane, Australia; 2 Malaria, Other Vectorborne and Parasitic Diseases, Regional Office for the Western Pacific, World Health Organization, San Lazaro Hospital Compound, Manila, Philippines; 3 National Vector Borne Disease Control Programme, Ministry of Health, Honiara, Solomon Islands; 4 National Vector Borne Disease Control Programme, Ministry of Health, Port Vila, Republic of Vanuatu; 5 Statistics and Demography Programme, Secretariat of the Pacific Community, Noumea, New Caledonia; 6 Swiss Tropical & Public Health Institute, 4002 Basel, Switzerland & University of Basel, Basel, Switzerland

## Abstract

**Background:**

Geographical Reconnaissance (GR) operations using Personal Digital Assistants (PDAs) and Global Positioning Systems (GPS) have been conducted in the elimination provinces of Temotu, Solomon Islands and Tafea, Republic of Vanuatu. These operations aimed to examine modern approaches to GR to define the spatial distribution of target populations to support contemporary malaria elimination interventions.

**Methods:**

Three GR surveys were carried out covering the outer islands of Temotu Province (October - November, 2008); Santa Cruz Island, Temotu Province (February 2009) and Tanna Island, Tafea Province (July - September 2009). Integrated PDA/GPS handheld units were used in the field to rapidly map and enumerate households, and collect associated population and household structure data to support priority elimination interventions, including bed net distribution, indoor residual spraying (IRS) and malaria case surveillance. Data were uploaded and analysed in customized Geographic Information System (GIS) databases to produce household distribution maps and generate relevant summary information pertaining to the GR operations. Following completion of field operations, group discussions were also conducted to review GR approaches and technology implemented.

**Results:**

10,459 households were geo-referenced and mapped. A population of 43,497 and 30,663 household structures were recorded during the three GR surveys. The spatial distribution of the population was concentrated in coastal village clusters. Survey operations were completed over a combined total of 77 field days covering a total land mass area of approximately 1103.2 km^2^. An average of 45 households, 118 structures and a population of 184 people were recorded per handheld device per day. Geo-spatial household distribution maps were also produced immediately following the completion of GR fieldwork. An overall high acceptability of modern GR techniques and technology was observed by both field operations staff and communities.

**Conclusion:**

GR implemented using modern techniques has provided an effective and efficient operational tool for rapidly defining the spatial distribution of target populations in designated malaria elimination zones in Solomon Islands and Vanuatu. The data generated are being used for the strategic implementation and scaling-up of priority interventions, and will be essential for establishing future surveillance using spatial decision support systems.

## Background

Following recent international attention, renewed political commitment and dedication of resources, the concept of malaria elimination and a refocusing of intensive malaria control is now back on the world agenda [1]. Recent global malaria maps have illustrated patterns of worldwide malaria endemicity, highlighting opportunistic areas for malaria elimination and intensified malaria control [2]. As part of this increased focus, current strategies for the reduction of the global burden of malaria and the eventual elimination of the disease have been outlined in the Roll Back Malaria (RBM) Global Malaria Action Plan (GMAP). These include: the scaling up and sustainment of intensive malaria control operations; progressively eliminating malaria from the endemic margins inward (i.e. shrinking the malaria map); and the continuation of research into new tools and approaches to malaria control and elimination [3,4].

To achieve the desired outcomes of the GMAP, it is essential that all of these strategies proceed concurrently [3]. As malaria control operations intensify and an increasing number of national programmes move towards the goal of elimination, the scaling-up of interventions, such as indoor residual spraying (IRS) and long-lasting insecticidal bed nets (LLIN), are essential. Similarly, as these interventions are rolled out, so will the need to adopt effective and practical operational tools for the coordination, monitoring and surveillance of key activities within target areas.

Geographical Reconnaissance (GR) involves census, mapping and sampling procedures to determine the quantity, location and accessibility of settlements within target areas. It provides the basis for the selection of field centres and depots, for designing schedules and itineraries of operations, planning deployment of transport, and assessing completion of planned activities [5]. GR has traditionally been used in malaria control and eradication programmes to identify target areas and enumerate populations for the coordination, implementation and quality control of operations. During the malaria eradication era of the 1950-60s, GR involved conducting detailed paper-based censuses and surveys of all households; sampling and measuring selected building structures; and developing locality-sketch maps of settlements using compass and pacing techniques [5,6]. GR can also be used to define as accurately as possible the geographical limits of malaria epidemics especially foci and assess epidemic potential [7,8].

Traditional GR operations required specialist skills, training and equipment in survey, navigation and cartography [5,6]. As such, a major disadvantage of traditional paper-based GR has been the time-consuming and resource-intensive processes involved, often limiting the willingness of programme managers to undertake such activities. However, with the growing availability of spatial data and access to geographic information system (GIS) technology, the applicability of mapping and spatial analysis in vector-borne disease management is now well established [9-20]. The use of Personal Digital Assistants (PDAs) and Global Positioning Systems (GPS) in the field has proven to be an accurate and cost-effective approach to the collection and geo-positioning of data at a household level [21-25]. Through the combination of baseline spatial data and integrated handheld PDA/GPS technology, GR can now be implemented more efficiently.

To support the global elimination strategy of shrinking the malaria map from the endemic margins inward [3], progressive elimination programmes in Solomon Islands and Vanuatu have been implemented with support from the Pacific Malaria Initiative (PacMI) programme, funded by the Australian Agency for International Development (AusAID). This programme aims to support the National Vector Borne Disease Control Programmes (NVBDCP) of the two countries to scale up intensified control nationwide and to eliminate malaria in selected provinces. The main interventions are universal household LLIN distribution and focal IRS. Elimination activities have commenced in Temotu Province in Solomon Islands and Tafea Province in Vanuatu [26].

To date, little research has addressed the approach, relevance and feasibility of GR within current-day malaria elimination frameworks. The aims of this study were to examine a modern approach to GR using digital geospatial survey technology and explore its application in contemporary malaria elimination programmes. To support this study, GR operations were conducted in the elimination provinces of the Solomon Islands and Vanuatu. Additional aims of these operations were to define and quantify the target populations and household structures; to provide a framework for the planning, implementation and monitoring of nominated elimination interventions; and to establish a foundation from which future malaria elimination surveillance activities can be carried out in these remote communities.

## Methods

### Survey area(s)

**Figure 1:**
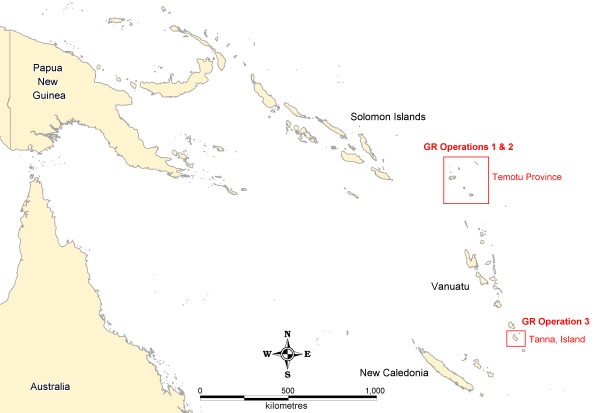
**General Location map of Geographical Reconnaissance Operation Areas**.

Baseline GR operations were conducted in the malaria elimination provinces of Solomon Islands and Vanuatu (Figure 1). Two separate surveys were carried out in Temotu Province, Solomon Islands, covering all islands except the remote Polynesian islands of Tikopia and Anuta, which are known to be malaria-free. Operations were conducted in the outer islands of Temotu from October-November 2008 (GR Operation 1), and on the main island of Santa Cruz during February 2009 (GR Operation 2). In Vanuatu, GR operations were conducted in the previously designated focal coastal IRS zone and northern health zone 2 of Tanna, Tafea Province, from July-September 2009 (GR Operation 3). Approval to conduct GR activities was provided by the Ministries of Health in each country, who formally requested support for these activities through in-country technical and development partners (i.e. WHO, PacMISC). Fieldwork was conducted by NVBDCP staff, with technical assistance from WHO and PacMISC.

### Development of spatial data collection systems

Selection of hardware and software was based upon the existing government systems and operational capacity within Solomon Islands and Vanuatu; practicality and durability of equipment in the field; and maximizing integration across all levels of data collection, management and analysis. Hardware included Trimble Juno ST (Trimble Navigation Limited, Sunnyvale, CA) handheld PDA units with integrated GPS and Durabook (GammaTech Computer Corp., Fremont, CA) notebook computers for data management and daily backup. ArcPad 7.0 (ESRI, Redlands, CA) software was used for data collection and field mapping operations, with post-fieldwork data storage, backup and analysis using both Microsoft Access (Microsoft Corporation, Redmond, WA) and MapInfo Professional 8.0 (Pitney Bowes Software Inc., Troy, NY). Otterbox casings were also used in the field to provide a protective cover for the handheld PDA/GPS units. Handheld PDA/GPS devices, accompanying software and protective casing were purchased prior to the GR operations, each collectively costing approximately $1500 per unit. All other hardware and software was sourced from existing Ministry of Health resources.

Collaborative agreements were developed between the NVBDCP and relevant Government GIS data custodians (i.e. the Ministry of Lands in both countries) to gain access and share data between the Ministries. These data were then adapted to produce standardized baseline topographic maps. To aid general reconnaissance throughout the household surveys, these maps were uploaded onto the handheld PDA units as underlying GIS-based ArcPad maps. These were used to provide a base from which households could be rapidly mapped, surveyed and viewed in the field in relation to surrounding features such as watercourses, coastlines, and local infrastructure.

**Figure 2:**
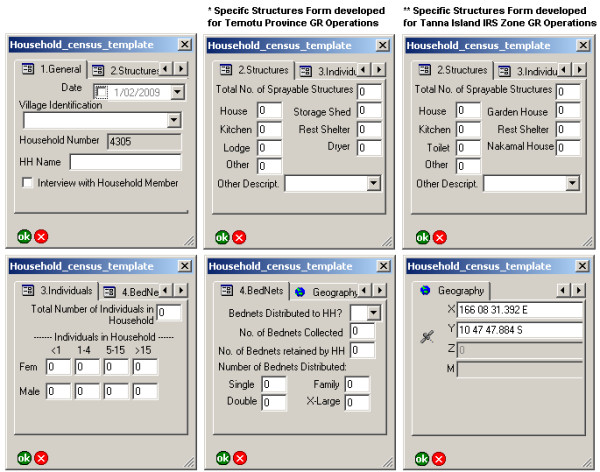
**Arcpad 7.0 Digital Data Collection Form developed for Geographical Reconnaissance Operations**.

Digital data collection forms (Figure 2) were developed in ArcPad in consultation with relevant stakeholders. These forms contained the following information:

i) Household identification and enumeration

▪ A unique identification number for every household

▪ Village name and name of the head of the household.

ii) The number, gender and ages of individuals residing in each household

iii) The number and types of structures (sleeping houses, kitchens, storage sheds, etc.) per household

iv) The number of mosquito nets currently in the household, and the number and sizes of new LLINs distributed (if relevant).

v) The geographical coordinates of the household (Projection: Longitude/Latitude WGS 84)

Automated numbers, checkboxes and drop-down menus were used in the design of the digital data collection form, to simplify and standardize data entry procedures. Separate forms were developed for Solomon Islands and Vanuatu due different traditional household structure types being dominant in these island groups.

**Figure 3:**
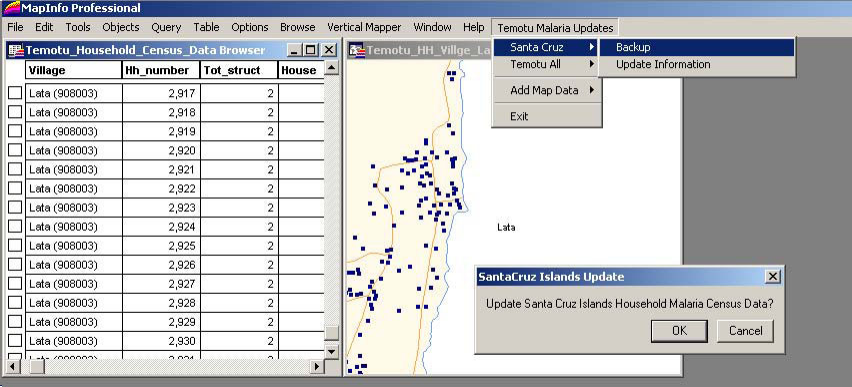
**Snapshot of automated GIS mapping application for Geographical Reconnaissance Operations**.

A customized GIS application was developed using MapBasic (Pitney Bowes Software Inc., Troy, NY) (Figure 3) to enable automatic backup of data from multiple handheld units into a central GIS data file stored on the field laptop, and to assist in daily data reviews, produce automated interactive household distribution maps and generate survey summaries to keep track of progress in the field.

### Planning, logistics and training

Meetings were conducted with relevant NVBDCP and community stakeholders prior to the implementation of GR to identify target areas, develop operational timelines and assist in resource allocation. Priority was placed on minimising human resources, by keeping field teams small and utilising community volunteers whenever possible. Six VBDCP field officers participated in the survey in the outer islands of Temotu Province, Solomon Islands, eight in the Santa Cruz, Temotu Province survey, and five in the Tanna Island, Tafea Province, Vanuatu survey. VBDCP officers participating in operations had no initial mapping or GR experience and were sourced from both national and provincial levels depending on the availability of personnel. In each field operation, survey teams were divided into individual survey mapping units, consisting of 2-3 individuals per unit (including community volunteers). Each mapping unit operated one handheld PDA and divided digital data entry, household mapping and household enumeration activities between the team. Field supervisors were also selected from the survey teams to be responsible for daily data backup, quality assurance and GPS data monitoring, and electrical charging of PDAs. Teams were supplied with handheld PDA/GPS units (including spare batteries), a field laptop computer, small printer, stationary and a portable generator. Prior to the commencement of each survey, workshops were conducted in Temotu and Tanna to train participating VBDCP personnel in the use of handheld PDA/GPS units. Additional training was also provided for field supervisors on daily data backup and quality assurance, data security, PDA troubleshooting, and mobile mapping and summary analysis techniques.

### Data collection and household mapping surveys

A standardized approach was applied across all three surveys. Following deployment to designated operation areas, field teams met with local community leaders to inform the community about the rationale of the survey. Local volunteers were also recruited from each village to act as guides and assist each unit in the household surveys. These volunteers served to increase community engagement, assist in language translation (when required), and provide local knowledge.

Because blanket LLIN distribution and focal IRS within each of these nominated target areas is to be implemented and monitored at the household level, the household was used as the primary unit for GR. Households in Melanesian countries are often associated with multiple separate structures including traditional sleeping houses, outdoor kitchens, resting shelters and storage sheds. To standardize GR operations, GPS coordinates were taken adjacent to the main sleeping house of each household. All other associated household structures were then identified, counted and recorded.

Cards were distributed as part of the household enumeration component to provide a hard-copy record of the household identity for future interventions. Survey teams (usually the community volunteer) also painted the unique household number on the top right-hand side of the household door or the nearest possible alternative. No issues with compliance associated with this activity were observed. Other structures (e.g. schools, churches and garden houses) were also mapped and enumerated as part of the survey and recorded as unoccupied structures.

Following the completion of daily household survey and mapping operations, PDA data were backed-up automatically onto the field laptop computers using the customised GIS application. Following daily data backup procedures, household distribution maps and survey summaries were also automatically updated using the customized GIS application to provide an overview of household distribution within the survey area and detailing daily progress. Household data quality assurance checks were also conducted by the field supervisor as part of the daily backup process to identify and remedy obvious digital data entry or GPS errors.

All household data were stored in central GIS-enabled databases located at the relevant national and provincial vector borne disease control programme offices of the elimination target areas. Data were automatically updated using the customized GIS application to link village, health operations zone, island, ward, and province names to each household unit. Using simple GIS techniques, descriptive maps of household distribution and summary statistics were then generated for elimination areas when required. Maps and household listings were printed in the field and given to local health facilities when relevant, providing a detailed overview of the spatial distribution of their health catchment populations.

### Assessing population at risk

GR data were overlayed onto existing malaria risk maps produced for Tanna, Tafea Province [27], to estimate populations residing in areas of above-average malaria prevalence. Overall, malaria prevalence on Tanna is estimated at 2.2% for *Plasmodium vivax *and 1.0% for *Plasmodium falciparum *[27]. Locations where malaria prevalence was above these respective overall prevalence rates were defined as having above-average risk. Households with a probability greater than 50% of having above-average risk were then identified and associated population and household structure data extracted. Population and household data were also summarized for the designated coastal IRS zone, covering all households located within 2 km of the coastline of Tanna.

### Assessment of modern geographical reconnaissance approach, technology and acceptability

Post-fieldwork interviews and debrief sessions where conducted with all participating VBDCP field officers and provincial managers following the completion of each GR operation. Informal sessions were held to ask participants about their individual perceptions of the GR procedures applied and associated PDA/GPS handheld equipment used in the field, and the relevance of this fieldwork for future elimination interventions. Participants were also asked to give insight on how to improve field operations using modern survey equipment. Group discussions were also held to provide feedback into the future improvements and potential applications of these techniques relevant to the malaria programme.

## Results

### Baseline data collection summary

A total of 10,459 households were geo-referenced and mapped across the two countries. Data were recorded on a total of 30,663 household structures and a population of 43,497. Table 1 provides a breakdown of the survey data by operation zone. Detailed breakdowns of population by age and gender (see Additional file 1: GR population summary), and household structures by type (see Additional file 2: GR structures summary) are also provided.

**Table 1:** Summary of data collected during Solomon Islands and Vanuatu Geographical Reconnaissance operations, 2009

GR Operation	Operation Zone	Total Population	Total Populated Households	Total Households	Total Structures
GR1:Outer Islands, Temotu Province	Duff Islands	556	118	158	399
	Reef Islands	5958	1179	1468	3746
	Utupua	1300	259	330	770
	Vanikolo	1602	308	399	938
	**GR1: Total**	**9416**	**1864**	**2355**	**5853**

GR2: Santa Cruz, Temotu Province	Santa Cruz East	3950	717	881	1873
	Santa Cruz West	8162	1563	1970	4046
	**GR2: Total**	**12112**	**2280**	**2851**	**5919**

GR3: Tanna IRS Zone, Tafea Province	Health Zone 1	5363	1064	1283	5214
	Health Zone 2	9395	1863	2196	7299
	Health Zone 3	3096	653	773	2923
	Health Zone 4	4115	866	1001	3455
	**GR3: Total**	**21969**	**4446**	**5253**	**18891**

### General population and household distribution

The population of Temotu Province, Solomon Islands was concentrated in coastal village clusters. With the exception of West Santa Cruz, road infrastructure in Temotu was non-existent and access to most settlements was limited to the sea and/or walking trails. The highest populations in the outer islands were recorded on the low-lying Reef Islands and concentrated in coastal village clusters on the main islands of Ngawa, Ngalo and Fenualoa. Populations in Utupua, Vanikolo and Duff Islands were limited to patchy coastal villages, with no settlements in the inland mountainous regions.

**Figure 4:**
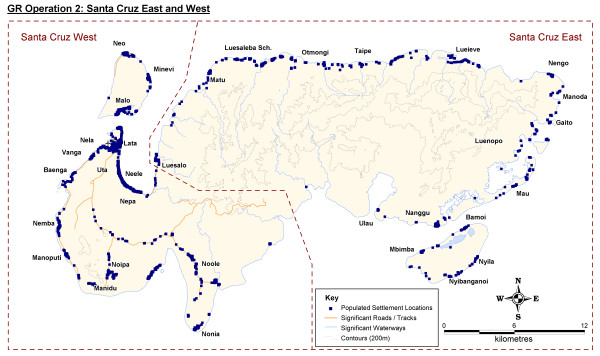
**Santa Cruz, Temotu Province, Solomon Islands Population Distribution Map**.

Population distribution on the most populous Temotu Province island group of Santa Cruz followed a similar pattern to the outer islands, with settlement clusters concentrated on the coastal fringes. However, settlements and household structures were also located within the low-lying inland regions of West Santa Cruz, where road infrastructure currently exists. Figure 4 provides an example island household distribution map produced as part of the Temotu Province, Solomon Islands GR operations.

**Figure 5:**
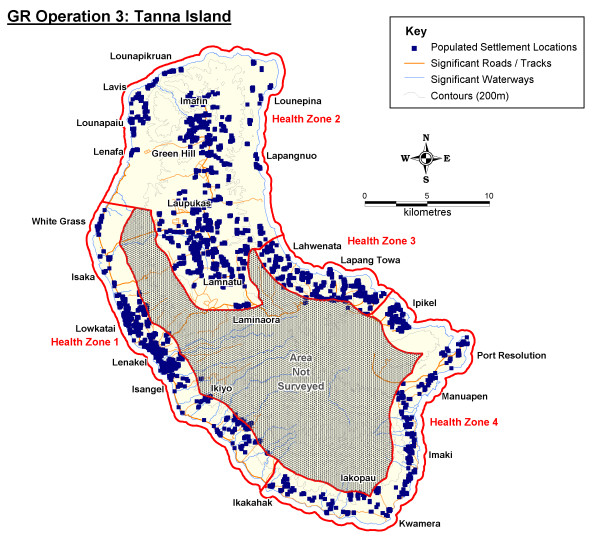
**Tanna GR Operation Zone, Tafea Province, Vanuatu Population Distribution Map**.

The population of the Tanna GR survey area in Tafea Province, Vanuatu, had greater spatial dispersion but was concentrated in coastal regions and highland plateaus (Figure 5). A more extensive road network also currently exists on Tanna in comparison to Temotu Province in the Solomon Islands.

Unoccupied structures, generally associated with agriculture (e.g. copra dryers, garden and storage sheds), were recorded throughout the study areas. Whilst not permanently inhabited, these structures are still considered significant, particularly in target areas identified for IRS, because people are known to sleep in them on an intermittent basis.

**Figure 6:**
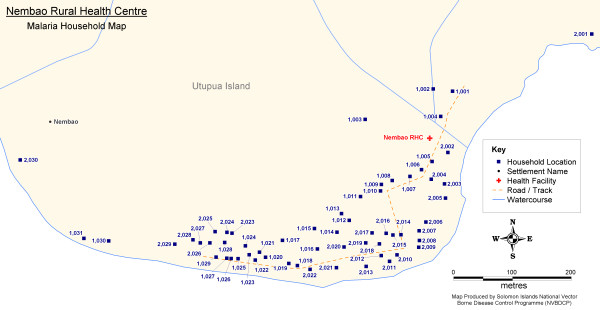
**Example Village Household Distribution Map**.

Figure 6 provides an example of a village household map produced in the field and presented to health facility officers in the remote outer islands of Temotu Province. (Note: Additional population distribution and operational maps can be made available upon request to the corresponding author)

### Efficiency of geographical reconnaissance field procedures

The three GR operations covered a land mass area of approximately 1103.2 km^2 ^and a total area of approximately 30,904.5 km^2 ^(including oceans). A total of 77 field days were required to complete operations for all three surveys, with an average of 45 households, 118 household structures and a population of 184 people recorded per PDA per day. Table 2 provides an overview of field procedures by individual GR operation. Average mapping times between each GR operation varied, with factors such as access, terrain, technical difficulties and level of field supervision all likely to have impacted field operations time. Technical difficulties arose in GR operation 2 - *Santa Cruz, Temotu Province*, where one PDA malfunctioned in a remote region and could not be repaired for three days, resulting in a moderate reduction of productivity in this area over this period. Some minor technical difficulties were reported during GR operation 3 - *Tanna IRS Zone, Tafea Province*. However, these were dealt with by the field-teams independently within one day following standardised trouble-shooting methods covered during initial training operations. No significant problems were recorded with GPS accuracy or digital data entry. No problems or data losses were also reported during the daily automatic backup procedures, with all team supervisors reporting favourably on all aspects of the daily automatic back-up, map verification and digital data entry checking procedures.

**Table 2:** Solomon Islands and Vanuatu Geographical Reconnaissance field procedures summary, 2009

GR Operation	Approx Land Area (km^2^)	Number of PDA/GPS units	Survey Days	Average Households Mapped (per PDA per Day)	Average Structures Recorded (per PDA per Day)	Average Population Recorded (per PDA per Day)	Technical Difficulties during Operation	Technical Field Supervision provided
GR1:Outer Islands, Temotu Province	79.63	2	19	61.97	154.03	247.92	No	Yes

GR2: Santa Cruz, Temotu Province	546.6	4	18	39.6	82.21	167.9	Moderate	No

GR3: Tanna IRS Zone, Tafea Province	476.97	4	40	32.83	116.28	136.74	Minor	Partial

### Acceptability of modern geographical reconnaissance approaches and technology

An overwhelmingly positive response to the modern GR approach and technology was expressed by both VBDCP field officers and provincial managers during post-operation interviews and group discussion. In particular, the time-saving benefits of no post-field work data entry requirements, the ability to map and visualise the spatial distribution of household data automatically, and the rapid generation of summary data was noted by provincial managers and information officers. Interest for applying GR techniques and mapping technology for other components of malaria field work such as entomological surveys, and for the expansion of these procedures into other malaria control provinces was also expressed. Additionally, the application of these techniques and technology to support other facets of provincial and regional health services was noted.

A high degree of community acceptability of modern mapping techniques was also observed in the field, with many community members taking a keen interest in the handheld units and associated GPS components during demonstrations in the field.

### The population at risk

Based on the Tanna Island risk maps, it was estimated that a population of 2,070 are residing in areas of above-average *P. falciparum *malaria prevalence, representing approximately 9.4% of the surveyed population. A total of 412 populated households, and 1,727 spray-able structures were estimated to be located in these focal areas. Similarly, it was estimated a total population of 3,782 reside in focal areas of above average *P. vivax *risk, representing 17.2% of the surveyed population. 713 populated households and 2,953 spray-able structures were also located in these areas. A total population of 13,818 and 12,453 spray-able structures were recorded in the designated coastal IRS intervention zone on Tanna, geo-referenced to a total of 3,365 individual household units.

## Discussion

To guide elimination interventions in these regions the NVBDCP of both countries highlighted a need for the collection of reliable, accurate and programme-specific data at a household level; particularly as relevant geo-spatial census data was unavailable at the level of precision required for the designated target elimination areas. Modern GR approaches and technologies were used to develop rapid and accurate field-based procedures for the collection, spatial definition and mapping of malaria elimination target populations. As these survey areas are located in remote Pacific islands, an emphasis was placed upon minimising the external resources (including human) required to undertake such operations and empowering local vector control programmes by introducing effective, user-friendly tools to support and efficiently guide the increased operational demands associated with malaria elimination.

In the context of the target elimination areas, the coastal concentration and isolation of populations without roads and reliable infrastructure, transportation and access are likely to be limiting factors to the success of elimination. Sound planning will be essential to ensure interventions are efficiently and effectively carried out across all target areas. Data compiled and collected during GR activities will enable programme managers to visualize the spatial distribution of populations to assist in the delineation of operational zones, the development of intervention timelines, and identifying transportation routes and access strategies. Relevant data pertaining to these priority interventions such as populations requiring LLINs and the total number of spray-able structures within focal IRS zones will assist in the accurate allocation of resources to designated operational zones. Household checklists and maps will also provide field officers with a detailed mechanism for conducting and monitoring priority interventions in target areas to ensure maximum coverage is achieved. Reporting will also be enhanced as the progress and coverage of interventions can be mapped and visualized at the household level.

Population and household structure results from GR operations carried out in the respective elimination zones illustrate the significant amount of detailed data that can be collected over large geographical areas by small teams and within short timeframes, highlighting the efficiency of modern approaches to GR. Whilst there is an increased need for pre-operation training when using the handheld units for data collection [21], the time and opportunity costs associated with training have been seen as valuable investments in building human resource capacity within the national malaria programmes of both countries. It is expected that as the technical competencies and experiences of field officers in the operation of these digital handheld devices increases, the lag time associated with troubleshooting technical difficulties in the field will be reduced. Since the completion of these initial GR operations, additional PDA/GPS based surveys have now been independently conducted by VBDCP officers in these regions and expanding into other provinces, reflecting the high acceptability, willingness and capacity of these programmes to now implement GR operations using these modern techniques and technologies.

Observations from the respective GR operations indicate a high willingness and capacity of VBDCP field staff to adopt and successfully utilize modern mobile mapping technology following initial basic training and technical guidance. The adoption of a simple customized GIS-based application to automate data back-up and mapping updates on a daily basis has also provided a rapid and effective mechanism for VBDCP staff to monitor progress and edit data easily in the field. As household mapping and PDA data entry operations were lead by VBDCP staff well known within their respective target communities, it is also likely this had an influence on the high level of community acceptability and compliance observed in the field, emphasising the importance of building local programme capacity to independently drive contemporary intervention strategies and approaches. Ethical approval was not sought for GR operations as they were considered routine operational activities of the national malaria programmes in both countries, with all collected data managed as per confidentiality requirements of the respective Ministries of Health.

Whilst some data entry errors are expected as a result of the digital data entry at the point of collection approach, previous research suggests such errors are minimal when compared to traditional paper-based surveys [22,24,25]. The cost-benefit of adopting handheld technology for field-based data collection has also been well established in earlier previous large-scale field research [21,22,24,25]. Relatively high-end handheld units were purchased prior to the implementation of these surveys with the anticipation of being used throughout all facets of elimination interventions. Whilst these specific units were somewhat expensive, they have been considered a viable investment as part of the respective national malaria programmes to support all routine field operations, mapping and data collection. With the growing availability of mapping and data collection software (including freeware and open source), as well as handheld and mobile phone technology capable of running GPS and data collection applications, alternative affordable technologies are also accessible to a wider market looking to implement GR principles.

Additional benefits such as the increased accuracy and resolution of GR data collected, and the immediate availability of summary information and maps relevant for priority elimination interventions also make integrated PDA/GPS handheld technologies favourable; overcoming constraining issues associated with traditional paper-based methodologies including low accuracy, and slow transaction times to verify data and prepare operational maps following fieldwork. The high portability of handheld PDA technology also provided considerable benefits in remote and difficult terrain where access and transportation posed significant logistical challenges.

In addition to the traditional applications of defining target populations and providing operational support, GR also provides an effective mechanism for further strengthening current-day priority monitoring and evaluation interventions such as detailed surveillance and case investigation. Malaria elimination requires robust and efficient surveillance mechanisms with full geographical coverage of target areas [28]. Active surveillance of high-prevalence foci is also essential for successfully interrupting malaria transmission [29]. The application of modern geo-spatial technology for GR can empower local programmes to carry-out detailed mapping and data collection operations in target areas efficiently and accurately. Data collected during the GR operations now provides a spatial framework to not only guide key interventions such as LLIN distribution and IRS, but also carry-out surveillance and investigation at a household level across entire populations living in elimination areas.

When coupled with additional tools such as malaria risk maps, entomology and mobility data, and local and historical knowledge of malaria transmission, strategic active surveillance can also be targeted and prioritized in key focal locations following GR operations. It is anticipated that the compilation of such data will provide the foundation for the establishment and expansion of spatial decision support systems (SDSS) in these elimination provinces. A SDSS developed from the ground up, offers the potential to provide a user-friendly tool to equip locally-based programmes in meeting the demands associated with the scaling-up of interventions and surveillance operations in malaria elimination as well as intensified malaria control regions.

## Conclusion

This study demonstrated a contemporary approach to GR to spatially define and enumerate target populations using modern geospatial survey technology. The focus was building inter-departmental capacity to independently carry-out GR operations to support priority interventions in the malaria elimination provinces in Solomon Islands and Vanuatu. This approach has provided an effective mechanism for rapidly and accurately defining target populations where these interventions have been scheduled to take place. Data collected and modern techniques adopted during GR have empowered local VBDCP programmes with effective decision making and operational tools to coordinate and monitor scaled-up elimination interventions with the goal of ensuring universal coverage, and providing a foundation for future surveillance activities. Following the high acceptability and success of these initial GR operations, both the Solomon Islands and the Republic of Vanuatu Vector Borne Disease Control Programmes are now dedicated to the expansion and further refinement of GR using modern mapping techniques and integrated PDA/GPS handheld technologies, illustrating the high acceptability of these approaches and providing a basis for the future expansion of a comprehensive and integrated SDSS to guide progressive malaria elimination in the pacific.

## Competing interests

The authors declare that they have no competing interests.

## Authors' contributions

Authors that participated in the conception of the study design were GK and JH. Field training was conducted by GK. Field research operations were coordinated by EH, GK, JN, WB, & WD. SP provided technical guidance and support. ACAC provided guidance on the scientific aspects of the study and provided detailed commentary on manuscript drafts. Additional scientific and technical guidance was provided by AV, JH & MT. Manuscript drafting was carried out by GK with support from all authors. All authors read and approved the final manuscript.

## Supplementary Material

Additional file 1 **Demographic description of the population of Solomon Islands and Vanuatu Geographical Reconnaissance operation areas, 2009**. Data provides a detailed demographic description by age and gender of the population recorded within each geographical reconnaissance operation area Click here for file

Additional file 2 **Household structures summary table of Solomon Islands and Vanuatu Geographical Reconnaissance operation areas, 2009**. Data provides a detailed breakdown of the number of structures recorded by type within each individual geographical reconnaissance operation area. Click here for file
