# Supplementary material for: Modern geographical reconnaissance of target populations in malaria elimination zones
Source: Malar J. 2010 Oct 20;9:289. doi: 10.1186/1475-2875-9-289 (PMC2974750; doi:10.1186/1475-2875-9-289)
Supplement: Additional file 1 — Demographic description of the population of Solomon Islands and Vanuatu Geographical Reconnaissance operation areas, 2009. Data provides a detailed demographic description by age and gender of the population recorded within each geographical reconnaissance operation area [file 1475-2875-9-289-S1.PDF]

Demographic description of the population of Solomon Islands and Vanuatu Geographical Reconnaissance operation areas, 2009

GR Operation	Operation Zone	< 1yrs		1-4yrs		5-15yrs		>15yrs		
		**Male**	**Female**	**Male**	**Female**	**Male**	**Female**	**Male**	**Female**	**Total**
GR1:Outer Islands, Temotu Province	Duff Islands	4	7	35	31	83	48	175	173	556
	Reef Islands	84	65	361	372	890	854	1534	1798	5958
	Utupua	15	16	85	82	213	190	355	344	1300
	Vanikolo	23	17	94	90	248	233	438	459	1602
	**GR1: Total**	**126**	**105**	**575**	**575**	**1434**	**1325**	**2502**	**2774**	**9416**
GR2: Santa Cruz, Temotu Province	Santa Cruz East	36	37	213	215	659	517	1109	1164	3950
	Santa Cruz West	77	87	402	380	1166	1045	2394	2611	8162
	**GR2: Total**	**113**	**124**	**615**	**595**	**1825**	**1562**	**3503**	**3775**	**12112**
GR3: Tanna IRS Zone, Tafea Province	Health Zone 1	19	14	307	301	1068	1070	1244	1340	5363
	Health Zone 2	93	97	719	649	1780	1669	2115	2273	9395
	Health Zone 3	17	17	178	186	638	584	685	791	3096
	Health Zone 4	34	34	277	248	871	772	895	984	4115
	**GR3: Total**	**163**	**162**	**1481**	**1384**	**4357**	**4095**	**4939**	**5388**	**21969**
