# Supplementary material for: Modern geographical reconnaissance of target populations in malaria elimination zones
Source: Malar J. 2010 Oct 20;9:289. doi: 10.1186/1475-2875-9-289 (PMC2974750; doi:10.1186/1475-2875-9-289)
Supplement: Additional file 2 — Household structures summary table of Solomon Islands and Vanuatu Geographical Reconnaissance operation areas, 2009. Data provides a detailed breakdown of the number of structures recorded by type within each individual geographical reconnaissance operation area. [file 1475-2875-9-289-S2.PDF]

Household Structures Summary Table of Temotu Province, Solomon Islands and Tafea Province, Vanuatu Geographical Reconnaissance Operation Areas, 2009

GR Operation	Operation Zone	Houses	Kitchens	Toilets	Lodges	Rest Shelters	Storage Sheds	Garden Houses	Dryers	Nakamal Houses	Other Structures	Total
GR1:Outer Islands, Temotu Province	Duff Islands	196	123	NR	24	3	22	NR	6	NR	25	399
	Reef Islands	1551	1160	NR	149	107	221	NR	154	NR	404	3746
	Utupua	339	234	NR	34	26	56	NR	2	NR	79	770
	Vanikolo	422	302	NR	17	30	84	NR	1	NR	82	938
	**GR1: Total**	**2508**	**1819**	NR	**224**	**166**	**383**	NR	**163**	NR	**590**	**5853**
GR2: Santa Cruz, Temotu Province	Santa Cruz East	855	615	NR	71	50	80	NR	98	NR	104	1873
	Santa Cruz West	1948	1319	NR	97	96	204	NR	87	NR	295	4046
	**GR2: Total**	**2803**	**1934**	NR	**168**	**146**	**284**	NR	**185**	NR	**399**	**5919**
GR3: Tanna IRS Zone, Tafea Province	Health Zone 1	1778	1001	988	NR	376	NR	1	NR	120	950	5214
	Health Zone 2	3123	1654	1345	NR	392	NR	26	NR	134	625	7299
	Health Zone 3	1202	593	404	NR	140	NR	0	NR	51	533	2923
	Health Zone 4	1310	806	670	NR	169	NR	6	NR	61	433	3455
	**GR3: Total**	**7413**	**4054**	**3407**	NR	**1077**	NR	**33**	NR	**366**	**2541**	**18891**

*NR: Structure type not individually classified during census operations following initial stakeholder collaboration and structure surveys within each target area.
